# Origins of food crops connect countries worldwide

**DOI:** 10.1098/rspb.2016.0792

**Published:** 2016-06-15

**Authors:** Colin K. Khoury, Harold A. Achicanoy, Anne D. Bjorkman, Carlos Navarro-Racines, Luigi Guarino, Ximena Flores-Palacios, Johannes M. M. Engels, John H. Wiersema, Hannes Dempewolf, Steven Sotelo, Julian Ramírez-Villegas, Nora P. Castañeda-Álvarez, Cary Fowler, Andy Jarvis, Loren H. Rieseberg, Paul C. Struik

**Affiliations:** 1 International Center for Tropical Agriculture (CIAT), Km 17, Recta Cali-Palmira, Apartado Aéreo 6713, 763537 Cali, Colombia; 2 Centre for Crop Systems Analysis, Wageningen University, Droevendaalsesteeg 1, 6708 PB Wageningen, The Netherlands; 3 United States Department of Agriculture, Agricultural Research Service, National Laboratory for Genetic Resources Preservation, 1111 South Mason Street, Fort Collins, CO 80521, USA; 4 German Centre for Integrative Biodiversity Research, Leipzig, Germany; 5 School of Geosciences, University of Edinburgh, James Hutton Road, Edinburgh EH9 3FE, UK; 6 CGIAR Research Program on Climate Change, Agriculture and Food Security (CCAFS), Km 17, Recta Cali-Palmira, Apartado Aéreo 6713, 763537 Cali, Colombia; 7 Global Crop Diversity Trust, Platz der Vereinten Nationen 7, 53113 Bonn, Germany; 8 Auckland University of Technology, 55 Wellesley St E, Auckland 1010, New Zealand; 9 Bioversity International, Via dei Tre Denari, 472/a, 00057 Maccarese, Italy; 10 United States Department of Agriculture, Agricultural Research Service, National Germplasm Research Laboratory, Building 003, BARC-West, Beltsville, MD 20705-2350, USA; 11 School of Earth and Environment, University of Leeds, Leeds, UK; 12 School of Biosciences, University of Birmingham, Edgbaston, Birmingham B15 2TT, UK; 13 The Biodiversity Research Centre, Vancouver, British Columbia, Canada V6T 1Z4; 14 Department of Botany, University of British Columbia, Vancouver, British Columbia, Canada V6T 1Z4; 15 Department of Biology, Indiana University, Bloomington, IN 47405, USA

**Keywords:** crop diversity, crop domestication, crop improvement, crop origins, food security, plant genetic resources

## Abstract

Research into the origins of food plants has led to the recognition that specific geographical regions around the world have been of particular importance to the development of agricultural crops. Yet the relative contributions of these different regions in the context of current food systems have not been quantified. Here we determine the origins (‘primary regions of diversity’) of the crops comprising the food supplies and agricultural production of countries worldwide. We estimate the degree to which countries use crops from regions of diversity other than their own (‘foreign crops’), and quantify changes in this usage over the past 50 years. Countries are highly interconnected with regard to primary regions of diversity of the crops they cultivate and/or consume. Foreign crops are extensively used in food supplies (68.7% of national food supplies as a global mean are derived from foreign crops) and production systems (69.3% of crops grown are foreign). Foreign crop usage has increased significantly over the past 50 years, including in countries with high indigenous crop diversity. The results provide a novel perspective on the ongoing globalization of food systems worldwide, and bolster evidence for the importance of international collaboration on genetic resource conservation and exchange.

## 1. Introduction

Over a century ago, advances in botany, linguistics, phytogeography and genetics made it possible to begin to identify the geographical origins of food crops [1]. Building on this work, and informed by extensive travels over five continents, the Russian scientist N. I. Vavilov proposed a number of independent ‘centres of origin’ of cultivated food plants around the world, where he saw a diversity of traditional varieties of a wide range of crops, alongside their wild relatives. These putative centres of origin included Central America and Mexico; parts of the Andes, Chile and Brazil–Paraguay; the Mediterranean; the Near East; Ethiopia; Central Asia; India; China; and Indo-Malaysia [2–4].

Vavilov's interest in the centres of origin of crops was practical, as these regions were postulated to hold tremendous genetic variation that could be useful to the improvement of agriculture. Such variation was the product of adaptation of plants over relatively long periods of time to diverse environments and cultural practices. In these regions, for example, he hoped to find early-maturing varieties suitable for northern latitudes, and disease-resistant forms providing a solution to the mass starvation caused by cyclical failures of the wheat crop [5].

Since Vavilov, the regions of origin and diversity of different crops have been debated, investigated and refined, benefiting from an expanding body of archaeological, linguistic, genetic and taxonomic information [6–15]. ‘Centres of diversity’ came to be preferred over ‘centres of origin’, to account for the understanding that high concentrations of crop varieties and related wild species are not in every case located precisely where crops were initially domesticated [13]. The radiation of crops from their primary centres of diversity has also been more extensively documented, including identification of ‘secondary centres of diversity’ and other designations of more recent patterns of diversification for some crops (e.g. *Phaseolus* bean in Southwestern Europe [16], and barley [17] and oat [18] in North America).

Research into the origins and patterns of diversity of food plants has contributed to an appreciation that specific geographical regions, for example the Near East, have been of particular importance to the development of agricultural crops and thus to the evolution of human culture [11]. Yet despite the growing body of literature on the regions of diversity of food plants, their relative contributions to modern agriculture and the current human diet have not been quantified.

Here we determine the geographical origins of the agricultural crop species important in the food supplies (measured in calories, protein, fat and food weight) and the agricultural production systems (measured in production quantity, harvested area and production value) of countries worldwide. We analyse ‘primary regions of diversity’, which we define as areas typically including the locations of the initial domestication of crops, encompassing the primary geographical zones of crop variation generated since that time, and containing relatively high species richness in crop wild relatives. This analysis permits a novel estimation of the degree to which crops used in countries are native versus foreign in origin. To accomplish this, we estimate the degree to which countries produce and/or consume crops from primary regions of diversity other than their own (‘foreign crops’), and determine changes in patterns of use over the past 50 years. We discuss the relative importance of primary regions of diversity of crops in the context of the globalization of food systems, and its conservation and policy implications.

## 2. Material and methods

### (a) Food supply and agricultural production data

We analysed the full set of food crop commodities reported in national food supply and pertinent national production data provided by FAO [19]: for food supply, calories (kcal capita^−1^ d^−1^), protein (g capita^−1^ d^−1^), fat (g capita^−1^ d^−1^) and food weight (g capita^−1^ d^−1^); for production, production quantity (tonnes), harvested area (ha) and gross production value (million US$). National food supply from plants represents: national agricultural food crop production plus imports, plus or minus food reserve changes over the survey period; minus exports, quantities used for seed, animal feed and in the manufacture of non-food products, and losses during storage and transport [19]. While food supply data count only crops contributing directly to human diets, production data for crops such as maize and soya bean are potentially inclusive of livestock and industrial uses as well as human food. In the production analysis, we also included agricultural crops indirectly contributing to human food supplies via livestock production (i.e. alfalfa, clover and vetch). Non-food (e.g. fibre for clothing) crops and animal product commodities were not included in the analysis. Plant commodities comprised the same crop species were aggregated into single categories representing the crop as a whole (e.g. values for sesame seed oil and sesame seed were combined). After aggregation, 53 crop commodities remained in food supplies data, and 132 crop commodities in production data (electronic supplementary material, table S1).

For current food supplies and production systems, we analysed data for each crop commodity per country over the most recent 3 years for which sufficient data were available (2009–2011). All (177) countries that consistently reported during the time period were included for food supply variables, as well as for production quantity and harvested area (electronic supplementary material, table S2), covering 98.5% of the world's population. All (141) countries that reported production value data (current million US$) were included, covering 94.1% of the world's population [19].

For the analysis of changes in use of foreign crops over time, food supply data were assessed for each year from 1961 to 2009, and production data for each year from 1961 to 2011. In order to align all time periods and include as much of the world's population as possible, the current countries formerly comprising the USSR, Yugoslav SFR, Ethiopia PDR and Czechoslovakia were aggregated into their former countries, with national data summed per year for production measurements, and merged by weighted average based upon the population of the respective states during the respective reporting year for per capita food supplies measurements. Belgium and Luxembourg were reported together during 1961–1999, and therefore recent years listing the countries separately were merged as above. The remaining 152 comparable countries in the food supplies data matrix comprised 98.0% of the world's population [19]. The remaining 182 countries in the production quantity and harvested area data matrices comprised 99.7% of the global population, and the 115 countries remaining in the (constant 2004–2006 million US$) production value data matrix comprised 88.5% (electronic supplementary material, table S2) [19].

### (b) Geographical regions

Regions were delineated following national borders in order to form manageable units for the assignment of primary regions of diversity of all crops, and at a scale enabling comparison with national food supply and production data. Regional classifications followed those listed in annex 2 of the FAO *State of the world's plant genetic resources for food and agriculture* [20], modified to more accurately represent ecogeographical parameters driving plant species distributions. Specifically, both western and eastern Europe were split into north and south regions to account for cold temperate versus Mediterranean ecologies; Australia and New Zealand were segregated from the remaining (tropical) islands of the Pacific region, and South America was split into Andean, temperate and tropical regions. A total of 23 regions were delineated worldwide (electronic supplementary material, figure S1). To account for ecogeographical variation within countries and to minimize overestimating their use of foreign crops, those countries whose boundaries included more than one region were included in all appropriate regions (e.g. Colombia was assigned both to Andean and to tropical South American regions; electronic supplementary material, table S2).

Primary regions of diversity were assigned to crops based on published studies regarding origins and centres of crop diversity and species richness of closely related wild plants [6–14,21–27]. The primary region of diversity unit was chosen for this analysis based on its greater overall applicability across the literature in comparison with more precisely proposed centres of origin or centres of diversity. To be inclusive with regard to primary regions of diversity of crops, and to minimize overestimating countries' use of foreign crops, those crops whose primary diversity encompassed more than one designated region were listed in all appropriate regions (e.g. wheat was listed in Central Asia, West Asia and the South and East Mediterranean owing to the high diversity of traditional crop varieties and wild relatives in each of these regions) [12,14]. Forty-two of the total 53 crop commodities treated in food supplies data, and 116 of the total 132 crops in production data, were attributable to primary regions of diversity. The remaining commodities that were not clearly recognizable as specific crop species (e.g. ‘fruits, other’) were listed as ‘not specified’ (electronic supplementary material, table S1).

For each country, we determined the importance of each primary region of diversity around the world to its current (2009–2011) national food supply and national agricultural production by grouping consumed/produced crops for each variable by their primary regions of diversity. We constructed circular plots displaying these data with consuming/producing countries aggregated to the regional level, using code adapted from Abel & Sander [28]. Regional food supply values (kcal or g capita^−1^ d^−1^) were calculated by deriving a population-weighted average of national food supply values across countries comprising each region. Regional agricultural production values were calculated by summing national production values across countries comprising each region.

### (c) Use of foreign crops

We estimated the degree to which a country's food supply and national agricultural production system uses ‘foreign’ crops by determining the extent to which the supply/production system is composed of crops whose primary regions of diversity do not coincide at all with the region(s) within which that country is located. The method starts with the assumption that all crops within a given country's food supply/production system are foreign (100% use of foreign crops). The percentage contributions of any crops whose primary regions of diversity included the same region as the country were then subtracted to estimate a ‘maximum use’ of foreign crops metric per country. In this metric, those crop commodities whose regions could not be specified were assumed to be entirely of foreign primary regions of diversity. The sum of the percentage contribution of these non-specified crop commodities was then subtracted, resulting in a ‘minimum use’ of foreign crops metric, which assumed that the primary regions of diversity of all non-specified crop commodities included the same region as the country (modified from [21]). For example, 1163.7 kcal capita^−1^ d^−1^ (48.5%) of Mexico's total 2400.3 kcal capita^−1^ d^−1^ (mean 2009–2011) pertained to crops of Central America and Mexican primary regions of diversity, resulting in a maximum use of foreign crops value of 51.5%. Given that 174.7 kcal capita^−1^ d^−1^ (7.3% of total) of the food supply could not be specified to primary regions of diversity, under the assumption that these foods also pertained to Central America and Mexican primary regions of diversity, Mexico's minimum use of foreign crops value was 44.2%.

Mean use of foreign crops in the food supply and agricultural production system per country was estimated using an interval censoring method [29], where the response variable (the calculated use value in each country in each year) was bounded by the minimum and maximum estimates for each observation. This model allows the uncertainty around the true use value to be retained in the model-estimated coefficients. For estimates of current use of foreign crops, we modelled the mean of the most recent 3 years (2009–2011). For estimates of change in use from 1961 to 2009/2011, intercepts and slopes per country were modelled as random effects, where the mean hyperparameter for the random slopes represents the estimated slope (change in use over time) across all countries. We allowed a correlation between country-level intercepts and slopes, as countries with high usage of foreign crops have weaker use–time relationships than countries with low usage [30]. We present the estimates of usage in the first (1961) and last (2009/2011) year for which data are available; these were estimated as derived parameters based on the predicted value of use in each year. The interval-censored models were implemented using a Bayesian framework in JAGS (v. 3.4.0) called from R (v. 3.1.1), using the packages rjags and R2jags. Non-informative (‘flat’) priors were used for all coefficients. Convergence was assessed using the Gelman–Rubin diagnostic [31]. Foreign crop usage values reported in the text represent the mean of the posterior distribution for each parameter, ± the standard deviation. Credible intervals for each parameter are reported in electronic supplementary material, tables S6–S8.

## 3. Results

### (a) Countries use crops from multiple primary regions of diversity worldwide

**Figure 1.**
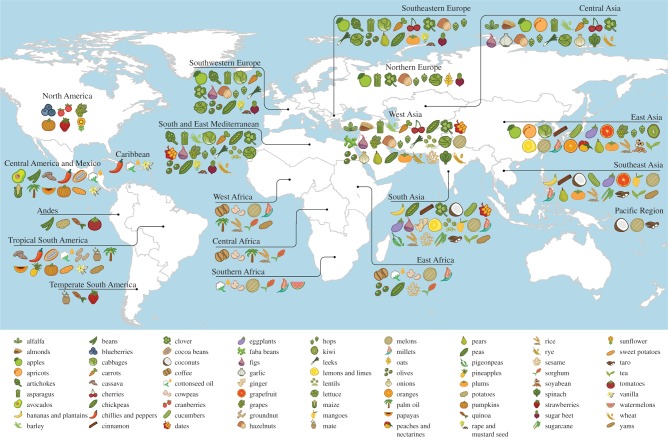
Primary regions of diversity of major agricultural crops worldwide. See electronic supplementary material, table S1 for a list of primary regions for all assessed crop commodities.

Primary regions of diversity of agricultural crops were identified across the world's tropics and subtropics, extending into temperate regions in both hemispheres (figure 1; see electronic supplementary material, figure S2 for a richness map of primary regions worldwide).

**Figure 2.**
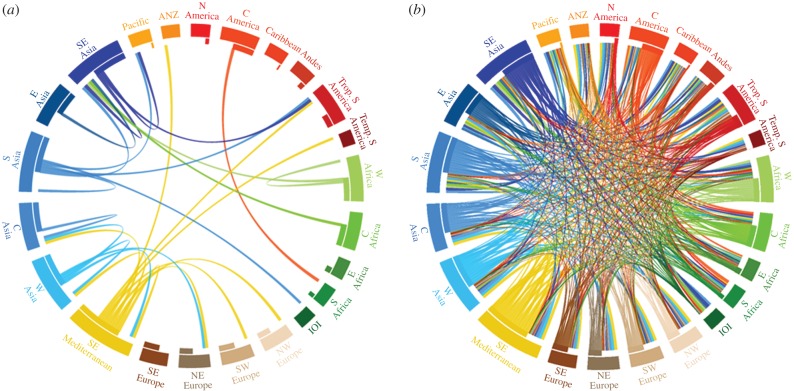
Circular plots linking the primary regions of diversity of food crops with their current importance in the context of calories (kcal capita^−1^ d^−1^) in regional food supplies. Each region has a colour representing its own native crops and those colours are connected to other regions due the importance of those crops in the food supply in other regions. The direction of the contribution is indicated by both the primary region's colour and a gap between the connecting line and the consuming region's segment. The magnitude of contribution is indicated by the width of the connecting line. Regional food supply values (per capita day^−1^) were formed by deriving a population-weighted average of national food supply values across countries comprising each region. IOI, Indian Ocean Islands; ANZ, Australia and New Zealand; C America, Central America and Mexico. (*a*) only the most significant linkages (i.e. 95th percentile) between regions are shown, for visibility, whereas (*b*) displays the full matrix of linkages. As an example, C America is represented in orange. The orange lines represent the amount of regional food supplies derived from crops native to the region—such as maize, beans and cassava—eaten in different regions of the world (see line connecting to Southern Africa owing to the high importance of these crops in that region). In turn, C America consumes crops native to other regions, for example, rice, coffee and sugarcane. See electronic supplementary material, figure S3 for circular plots for all measured food supply and production variables.

Food supplies and agricultural production systems worldwide were found to be composed of a wide range of crops deriving from several different primary regions of diversity, indicating a thoroughly interconnected global food system with regard to the geographical origins of food plants (figure 2; see electronic supplementary material, figure S3 for all measured variables, and electronic supplementary material, tables S3 and S4 for values per country and per region). Without exception, regional food supplies and agricultural production systems were linked to the majority of the world's primary regions of diversity owing to the extensive production and/or consumption of crops from different geographical regions. An interactive resource for exploring links between regional food systems and the primary regions of diversity is available at http://blog.ciat.cgiar.org/origin-of-crops.

The widespread importance in food supplies worldwide of crops such as wheat, rice, sugarcane, maize, soya bean, potatoes, barley, oil palm, beans, tomatoes, bananas and plantains, and sugar beet, among others, led to the particular significance of West, Central, South, Southeast and East Asian, South and East Mediterranean, West and Central African, Central American and Mexican, Andean and tropical South American, and southern European primary regions of diversity (figure 2; electronic supplementary material, figure S3; see in particular the quantity of lines originating from these regions and connecting to most other regions in these figures; also see electronic supplementary material, figure S4 for a depiction of the relative importance of primary regions of diversity in contribution to global aggregate food supply and total global agricultural production, and electronic supplementary material, table S5 for values per crop per region).

At the national level, primary regions of diversity of greatest importance in the context of calories, protein and food weight included Central America and Mexico, the South and East Mediterranean, West, Central, South, East and Southeast Asia, and tropical South America (electronic supplementary material, table S3). In Malawi, for example, the primary regions of diversity identified as of greatest importance in the context of calories included Central America and Mexico (for crops including maize, cassava and beans), South and Southeast Asia (sugarcane, rice, and bananas and plantains), the South and East Mediterranean (wheat), tropical South America (cassava and groundnut), and Andean South America (potatoes and beans).

The primary regions of diversity of greatest importance for fat in national food supplies included East Asia (owing to the significance of soya bean in many regions), Central and West Africa (palm oil, in countries in these regions as well as in Central American and Southeast Asian nations), tropical South America (groundnut and palm oil, particularly in West and Central African countries), North America (sunflower in Eastern European countries), Central America and Mexico (maize in southern African countries), South and Southeast Asia (coconuts in Southeast Asian and Pacific Region countries), and southern Europe (rape and mustard, particularly in European and North American countries). South and Southeast Asia, Central America and Mexico, Andean South America, West, Central and East Asia, and the South and East Mediterranean were the primary regions of diversity of agricultural crops of greatest overall importance to national agricultural production metrics.

### (b) Countries make substantial use of foreign crops

Countries' use of foreign crops—plants whose primary regions of diversity do not coincide at all with the same regions within which the country is located—was extensive worldwide, both in food supplies and national agricultural production. Mean use of foreign crops across all countries in food supplies was (mean ± s.d.) 65.8% ± 1.8 for calories, 66.6% ± 2.1 for protein, 73.7% ± 1.6 for fat and 68.7% ± 1.4 for food weight. Mean cultivation of foreign crops in national production systems was 71.0% ± 1.8 for production quantity, 64.0% ± 2.2 for harvested area and 72.9% ± 1.9 for production value. The combined mean use of foreign crops across food supply variables was estimated at 68.7%, across production systems at 69.3%, and across food systems worldwide (i.e. across all countries and all food supplies and agricultural production variables) at 69.0% (electronic supplementary material, table S6).

**Figure 3.**
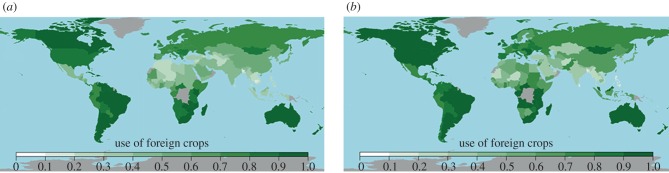
Use of foreign crops per country. Maps display the degree of (*a*) calories in national food supplies and (*b*) quantity (tonnes) in national agricultural production that are derived from crops whose primary regions of diversity do not coincide with the same region as the country (foreign crops). Scale is degree of foreign crop use (1 = 100% use of foreign crops). As an example, (*a*) demonstrates that the calories consumed in Canada (dark green) are highly derived (estimated value is 92.5% ± 2.6) from crops whose primary regions of diversity do not include the North America region. See electronic supplementary material, figure S5 for world maps displaying foreign crop use per country for all measured food supply and production variables.

Use of foreign crops in national food supplies and production systems was highest (i.e. up to 100%) in countries that were geographically isolated, and/or located at great distance from the primary regions of diversity of major staple crops (figure 3; see electronic supplementary material, figure S5 for all measured variables, and electronic supplementary material, table S6 for values per country). This included Australia and New Zealand, the Indian Ocean Islands, the Caribbean, southern South America, North America, southern Africa and northern Europe. These countries are generally in temperate climates, although tropical islands and some continental tropical regions, such as Central Africa, also demonstrated very high levels of use of foreign crops for most variables.

Conversely, use of foreign crops was lowest in countries located within the primary regions of diversity of major crops, and where traditional staples are still cultivated and consumed, such as Southeast Asia, the South and East Mediterranean, South Asia, Central Asia, West Asia and West Africa. The lowest levels of foreign crop use (e.g. 19.1% ± 0.7 in Cambodia, 20.0% ± 0.7 in Bangladesh and 20.1% ± 0.8 in Niger, for calories) were found in countries with food systems dominated by a limited number of traditional staples such as rice, wheat, yams, sorghum and millets. Island nations predominantly consuming native crops for fat (e.g. coconut in the tropical Pacific Region) and countries with extreme agroecological conditions limiting national production to the cultivation of a select number of native crops (e.g. dates in the United Arab Emirates) also exhibited very low levels of foreign crop use for relevant food supply or production metrics. In such extreme cases, though, low use of foreign crops was generally evident in only one or a few food supply or production metrics, whereas other variables exhibited much higher usage.

Although food supplies and agricultural production variables were well correlated in degree of foreign crop use (electronic supplementary material, figures S6 and figure S7), variation was also visible across variables, with highest overall use evident in fat, production value, production quantity and food weight (electronic supplementary material, table S6).

### (c) Countries' use of foreign crops has increased over time

**Figure 4.**
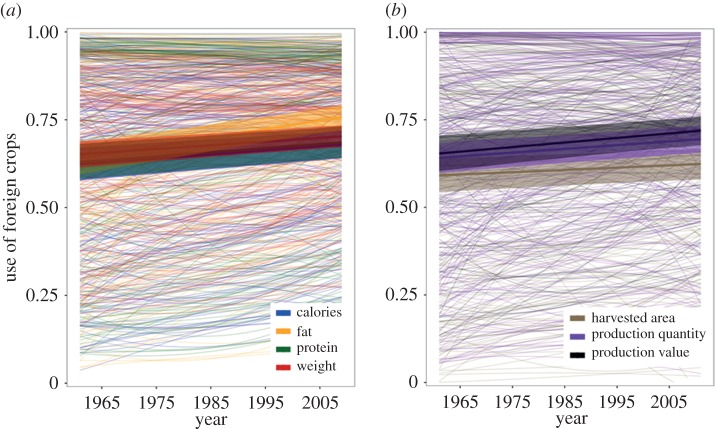
Change in use of foreign crops in (*a*) national food supplies from 1961 to 2009, and (*b*) national agricultural production from 1961 to 2011. Lines represent change over time for each country in each year for each variable. Transparent ribbons represent modelled mean change across all countries (± 95% credible interval). See electronic supplementary material, figure S8 for world maps displaying slopes of change in foreign crop usage per country for all measured food supply and production variables.

Use of foreign crops by countries increased significantly as a global mean for all food supply and agricultural production variables over the past half-century (figure 4; see electronic supplementary material, tables S7–8 for values per country per year). Foreign crop use with regard to calories increased from 62.2% ± 2.4 to 67.8% ± 2.0, protein from 62.9% ± 2.6 to 68.5% ± 2.2, fat from 63.8% ± 2.3 to 75.5% ± 1.8 and food weight from 65.1% ± 1.9 to 70.2% ± 1.6 from 1961 to 2009, averaged across countries worldwide. Likewise, foreign crop cultivation in terms of production quantity increased from 64.2% ± 2.2 to 69.5% ± 2.0, harvested area from 59.2% ± 2.5 to 62.4% ± 2.3 and production value from 65.4% ± 2.6 to 71.9% ± 2.1 between 1961 and 2011.

Countries with the largest increases in foreign crop usage over the period were located in Africa, West, South, Southeast and East Asia, Central America, and Andean and tropical South America (see electronic supplementary material, figure S8 for maps displaying slopes of change over time). A number of countries with the largest changes in use of foreign crops in contribution to their food supplies were also those with major transitions in their production systems during the past 50 years (e.g. the growth of oil palm cultivation in Malaysia and Indonesia, a crop whose primary regions of diversity are located in West and Central Africa and the Neotropics; and soya bean in Brazil, a crop of East Asian origin). Foreign crop use with regard to fat increased the most of all variables over the past 50 years, a trend that is concordant with significant changes in the crop species composition of plants in national food supplies globally over this period [32].

## 4. Discussion

The geographical isolation that contributed to the development of variation in cultivated food plants also largely restricted this diversity to its primary regions or nearby areas throughout most of recorded history, although notable long-distance migrations of some crops have been recognized (e.g. sorghum and millets between Africa and South Asia [33] and maize in the Americas [34]). The ‘age of discovery’ and in particular the Columbian Exchange marked key accelerations in the movement of food plants, as they were introduced to colonizing countries and to new regions with growing colonial establishments and emerging export-oriented production [35–37]. The movement of food crops during the Columbian exchange happened quite quickly for many of these plants. Potatoes, for example, were first seen by European explorers in 1551 and were already being cultivated in the Canary Islands in 1567 [38]. Cultivation in new agricultural areas was in many cases remarkably successful, in part owing to escape from crop-specific pests and pathogens [39]. Complementarity in terms of production season or dietary needs also facilitated some crops' rapid acceptance (e.g. maize in Italy) [40].

The expansion of human settlement to the limits of the inhabitable areas of the planet, driven by ever more efficient transportation and increases in global trade, have decoupled the geography of consumption of crops from their production [41]. Bananas, a crop requiring tropical growing conditions, are now consumed in at least 167 countries, including all temperate regions [19]. Ongoing economic and agricultural development and globalization trends, including increasing consumer purchasing power in developing regions, the rise of supermarkets and convenience foods, greater consumption outside the home, urbanization, refrigerated transport, agricultural subsidies, industrial food technologies and facilitated trade agreements, have made a greater variety of major food commodities available to consumers in countries worldwide, but in turn increased homogeneity in the global food system [32,42]. Given this homogenization in global food supplies, the geographical decoupling of agricultural production and food consumption [41,43–45], and greater consumption of packaged and processed food products [42], it is increasingly feasible to imagine not only mistakenly attributing the origin of potatoes to Ireland, tomatoes to Italy and chilli peppers to Thailand, but indeed losing the connection of crops with a geographical origin entirely.

Our results highlight the extensive connections among countries and regions worldwide with regard to the origins of crops important to their food supplies and agricultural production. The most important primary regions of diversity contributing to a country's modern food system are more often to be located elsewhere around the planet (figure 2*b*; electronic supplementary material, figure S3—note the significance of lines crossing the centre of the circle to connect different regions). While the persistence of traditional foods and perhaps even the traces of the original biological constraints on food plants are still visible in food supply patterns (e.g. figure 2*a*—wheat versus rice consumption by region is visible in the linking of West and Central Asia, South and East Mediterranean, and Europe; versus South, East and Southeast Asia, and West and Central Africa), the overriding trend is the considerable use of both native and foreign food crops, from both tropical and temperate primary regions of diversity. While the identification of key regions by Vavilov and subsequent authors as the birthplace of our most important foods is confirmed by the latest data, less well celebrated primary regions are also highly significant to modern food supplies and production systems, especially for fat (e.g. North America owing to sunflower, and West and Central Africa because of oil palm). We increasingly depend on each other's plants.

The interconnectedness of countries and regions with regard to primary regions of diversity of crops evident here is sufficiently pronounced so as to be robust to the use of different approaches to dealing with our source data. Nonetheless, a number of limitations and uncertainties should be noted. While the range of crops covered in this analysis encompasses all data currently available in globally comparable food supplies and agricultural production statistics recorded at the country level [19], the diversity of crops is not reported at high enough resolution to distinguish all contributing plant species. Therefore, an underestimation of diversity is likely, especially in countries with heterogeneous topographies, diverse cultures, and coarse agricultural and dietary intake reporting. This may be particularly the case for plants primarily encountered in home gardens and local markets, seasonally important foods, and culinary herbs, spices and other crops consumed in relatively small quantities [46]. The generality of the defined ecogeographical regions, subjectivity of the boundaries of the regions, and lack of knowledge for some crops as to their primary regions of diversity also contribute to ambiguity in linking food supplies and production systems to the regions of diversity of their crops and in deriving foreign crop usage metrics. Investments in country-level agricultural and food intake data and further research in the origins of food crops are needed for the completion of comprehensive analyses on the origins of total plant diversity produced and consumed in countries.

The extensive connections between countries and regions with regard to the primary regions of diversity of crops provide a novel perspective on the ongoing globalization of food systems worldwide. The increasing use of foreign crops bolsters the rationale for considering the underlying genetic diversity of important food plants as a global public good [47–49]. International agreements are justified to appropriately recognize historical and current contributions to the generation of this diversity, protect farmers' rights to choose what varieties they maintain and exchange, and promote the conservation and sustainable use of this crop genetic diversity [20,27,47–52]. Given the ongoing evolution of the global food system due both to dietary change [32,42] and increasing production challenges [53–56], a broadly inclusive effort to conserve and provide access to crop genetic diversity worldwide is prudent.

## Supplementary Material

Supplementary Material

## Data accessibility

Food supplies and production data from 1961 to the present are available from the FAOSTAT website at http://faostat.fao.org. The processed datasets supporting this article are described in the electronic supplementary material and are available on Dryad at http://dx.doi.org/10.5061/dryad.s08t2. Codes for building the circle diagrams and online interactive versions are available on GitHub at https://github.com/CIAT-DAPA/cwr_interdependence.
