# Supplementary material for: Origins of food crops connect countries worldwide
Source: Proc Biol Sci. 2016 Jun 15;283(1832):20160792. doi: 10.1098/rspb.2016.0792 (PMC4920324; doi:10.1098/rspb.2016.0792)
Supplement: Supplementary Material [file rspb20160792supp1.pdf]

Supplementary Material:

SI 1. Supplementary Figures

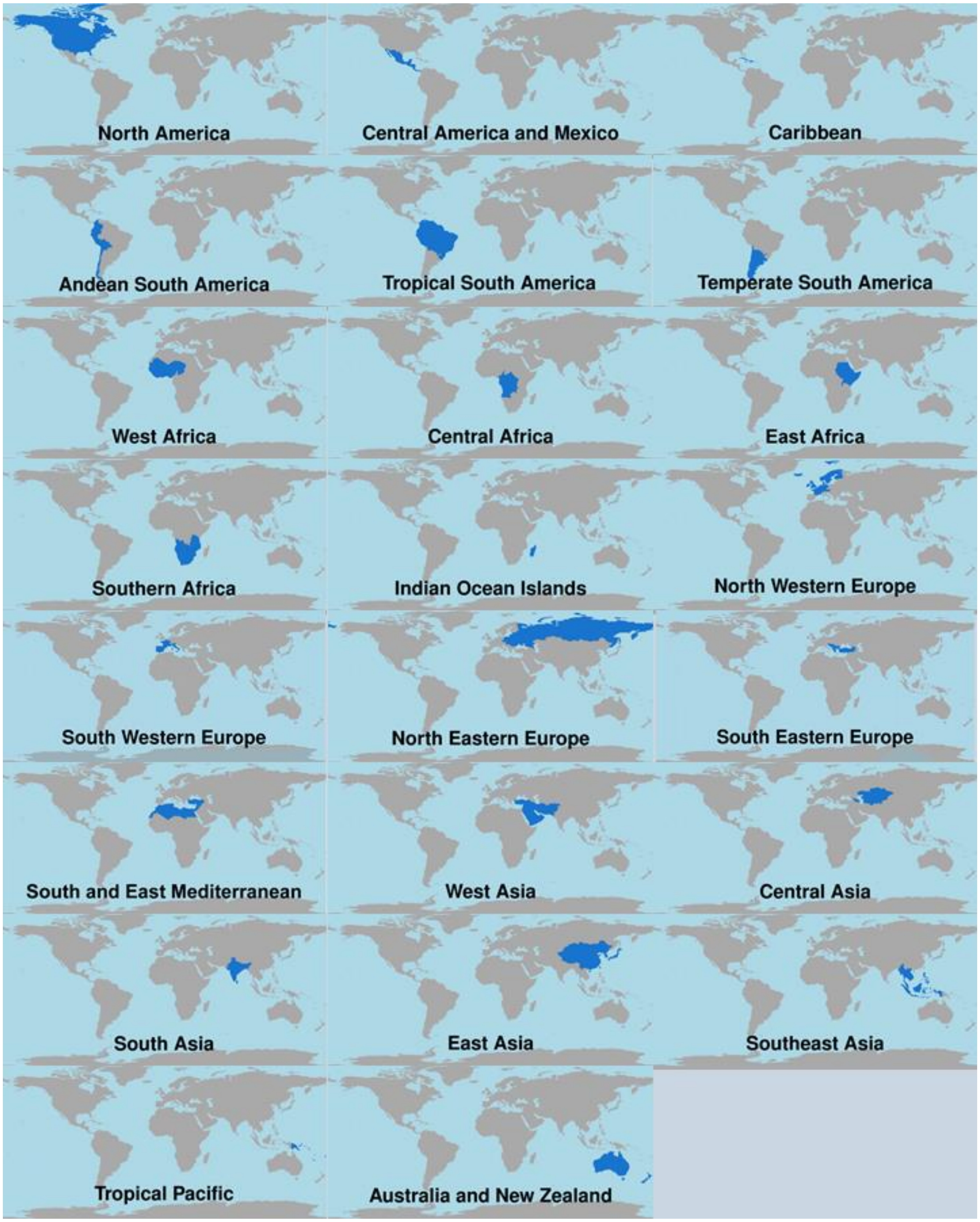

Figure S1. Eco-geographic regions utilized in the analysis. See Table S2 for a list of countries per region.

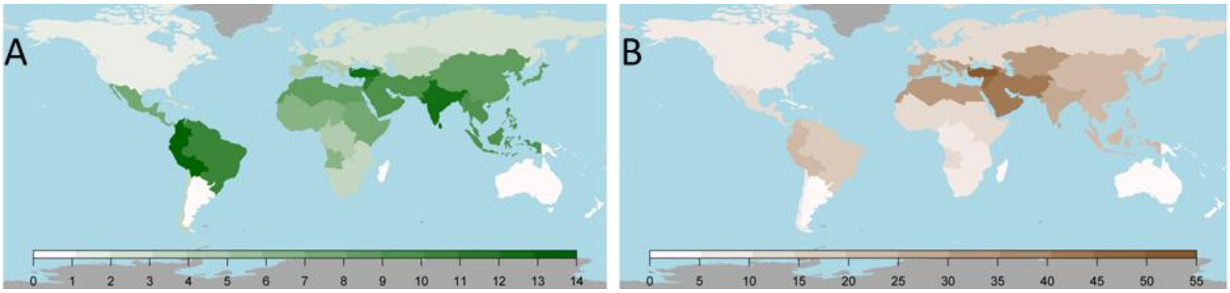

Figure S2. Richness maps of primary regions of diversity of crop commodities. (A) Primary regions of diversity of 42 crop commodities assessed in food supplies; and (B) Primary regions of diversity of 116 crop commodities assessed in agricultural production systems. Scale is number of crops; darker colors represent regions where numerous primary regions of diversity of crops overlap.

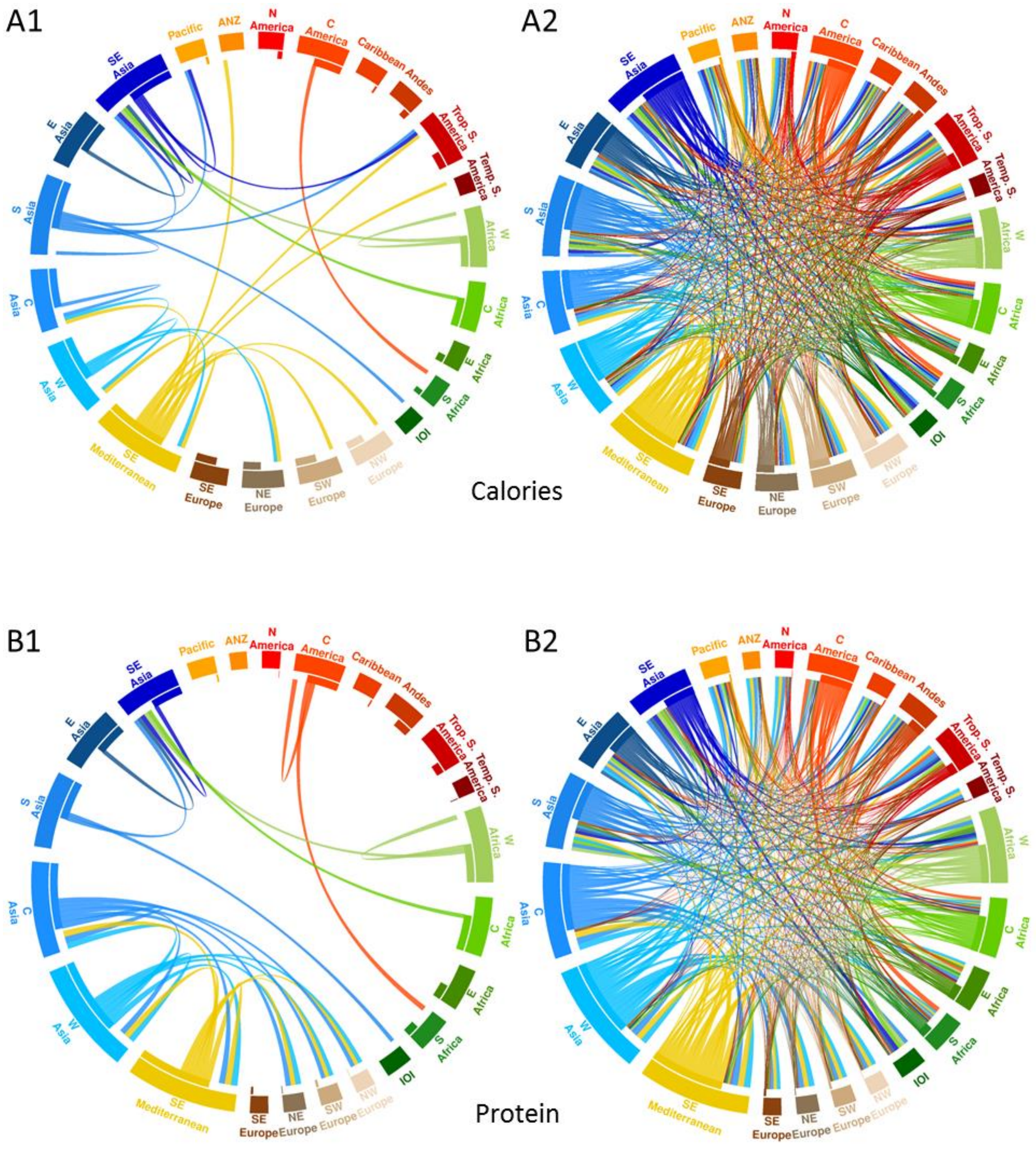
A1
A2
B1
B2
Pacific
ANZ
N America
C America
Caribbean
Andes
Trop. S. America
Temp. S. America
W Africa
C Africa
E Africa
S Africa
IOI
NW Europe
SW Europe
NE Europe
SE Europe
SE Mediterranean
W Asia
C Asia
S Asia
E Asia
SE Asia
Calories
Protein

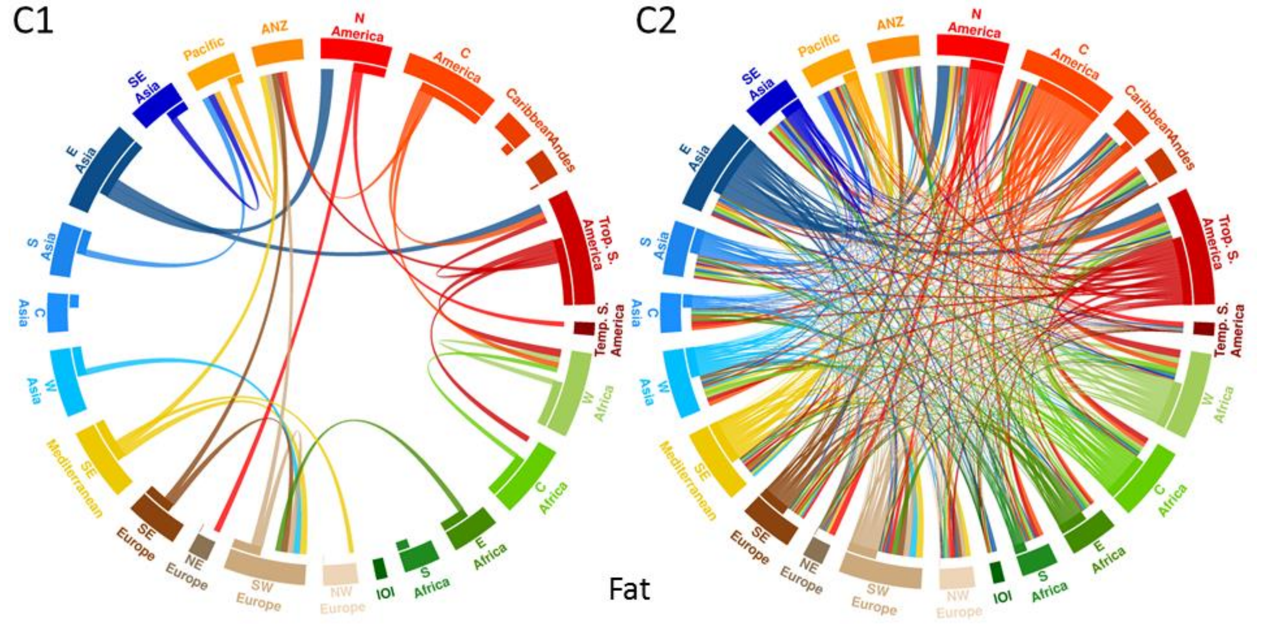
C1
N America
C America
Caribbean
Andes
Trop. S. America
Temp. S. America
W Africa
C Africa
E Africa
S Africa
IOI
NW Europe
SW Europe
NE Europe
SE Europe
SE Mediterranean
W Asia
C Asia
S Asia
E Asia
SE Asia
Pacific
ANZ
C2
N America
C America
Caribbean
Andes
Trop. S. America
Temp. S. America
W Africa
C Africa
E Africa
S Africa
IOI
NW Europe
SW Europe
NE Europe
SE Europe
SE Mediterranean
W Asia
C Asia
S Asia
E Asia
SE Asia
Pacific
ANZ
Fat

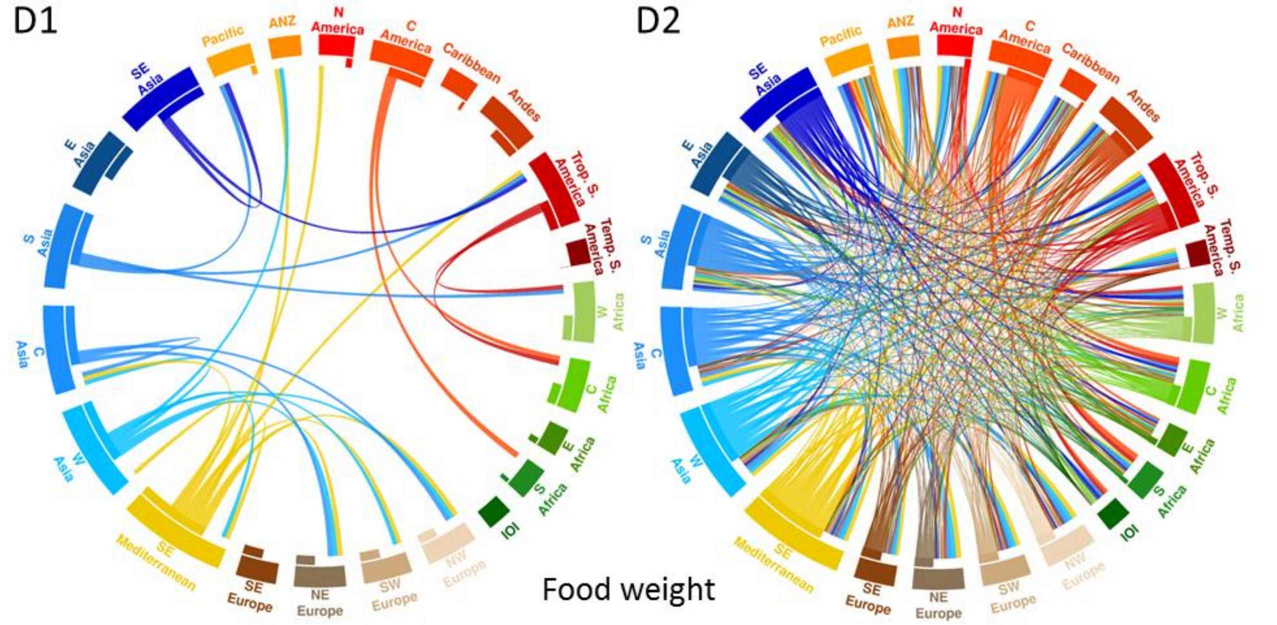
D1
N America
C America
Caribbean
Andes
Trop. S. America
Temp. S. America
W Africa
C Africa
E Africa
S Africa
IOI
NW Europe
SW Europe
NE Europe
SE Europe
SE Mediterranean
W Asia
C Asia
S Asia
E Asia
SE Asia
Pacific
ANZ
D2
N America
C America
Caribbean
Andes
Trop. S. America
Temp. S. America
W Africa
C Africa
E Africa
S Africa
IOI
NW Europe
SW Europe
NE Europe
SE Europe
SE Mediterranean
W Asia
C Asia
S Asia
E Asia
SE Asia
Pacific
ANZ
Food weight

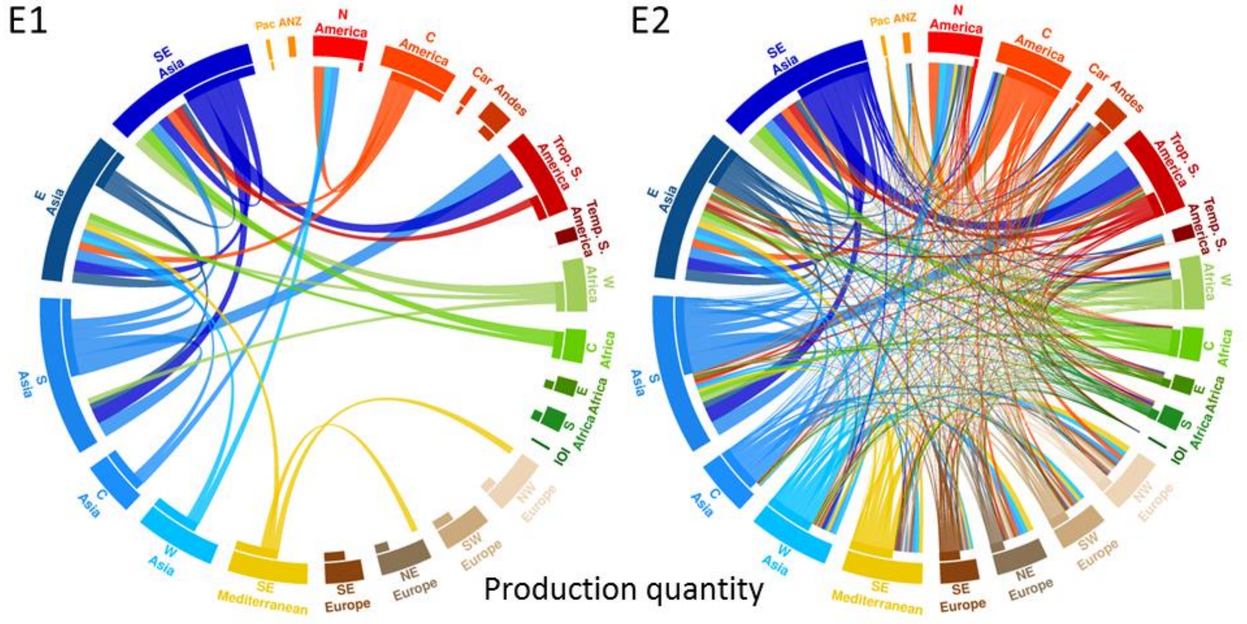
E1
E2
Pac
ANZ
N America
C America
Car
Andes
Trop. S. America
Temp. S. America
W Africa
C Africa
E Africa
S Africa
IOI
NW Europe
SW Europe
NE Europe
SE Europe
SE Mediterranean
W Asia
C Asia
S Asia
E Asia
SE Asia
Production quantity

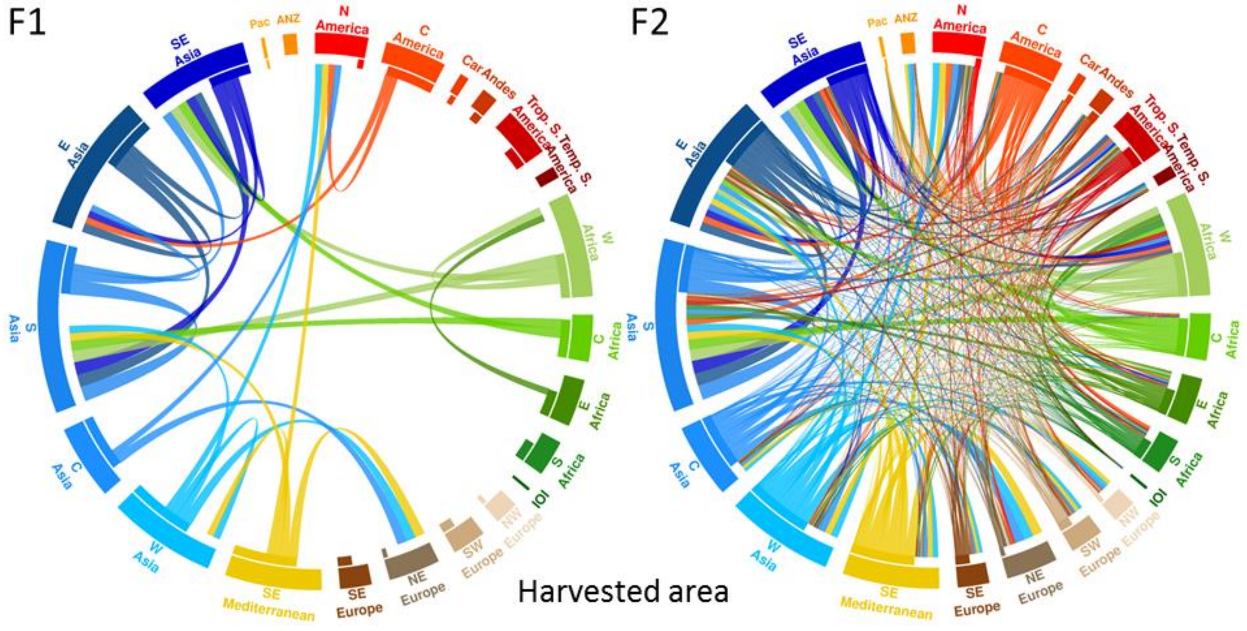
F1
F2
Pac
ANZ
N America
C America
Car
Andes
Trop. S. America
Temp. S. America
W Africa
C Africa
E Africa
S Africa
IOI
NW Europe
SW Europe
NE Europe
SE Europe
SE Mediterranean
W Asia
C Asia
S Asia
E Asia
SE Asia
Harvested area

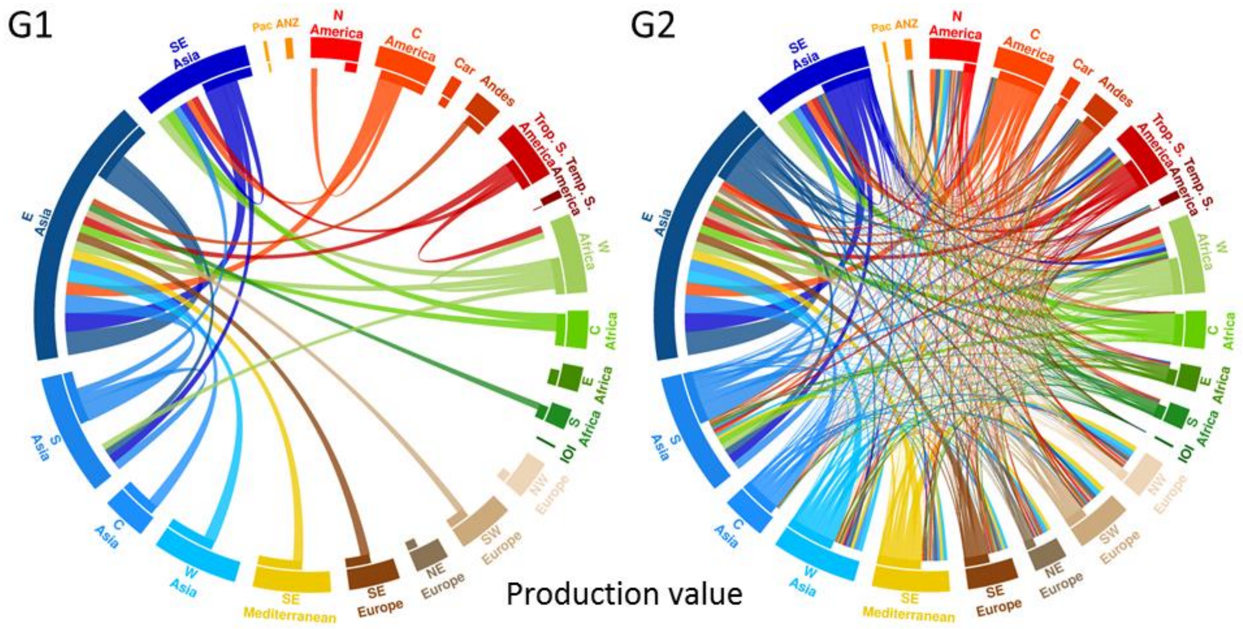

Figure S3. Circular plots linking the primary regions of diversity of food crops with their current (2009-2011 average) importance in regional food supplies, measured in terms of (A) calories (kcal/capita/day), (B) protein (g/capita/day), (C) fat (g/capita/day), and (D) food weight (g/capita/day),] and regional agricultural production systems, measured in terms of (E) production quantity (tonnes), (F) harvested area (ha), and (G) production value (current million US$). Each region has a color representing its own native crops and those colors are connected to other regions due the importance of those crops in the food supply/agricultural production in other regions. The direction of the contribution is indicated by both the primary region's colour and a gap between the connecting line and the consuming/producing region's segment. The magnitude of contribution is indicated by the width of the connecting line. Because the line width is nonlinearly adapted to the curvature, it corresponds to the contribution size only at the start and end points. Regional food supply values (per capita/day) were formed by deriving a population-weighted average of national food supply values across countries comprising each region. Regional agricultural production values were formed by summing national production values across countries comprising each region. For countries within regions, see table S2. Region names are shortened in the figures: IOI = Indian Ocean Islands, ANZ = Australia and New Zealand, and C. America = Central America and Mexico; and in production figures only, Car = Caribbean, and Pac = Tropical Pacific Region. Figures on the left (numbered 1) display only the most significant linkages (i.e., 95th percentile) for visibility; figures on the right (numbered 2) display the full matrix of linkages between primary regions of diversity in food supplies and agricultural production systems. As an example, tropical South America is represented in crimson. The crimson lines represent the amount of regional food supplies/agricultural production derived from crops native to the region- such as cassava, groundnut, and cocoa beans- eaten/produced in different regions of the world. In turn, tropical South America consumes/ produces crops native to other regions, for example, rice, sugarcane, and bananas and plantains.

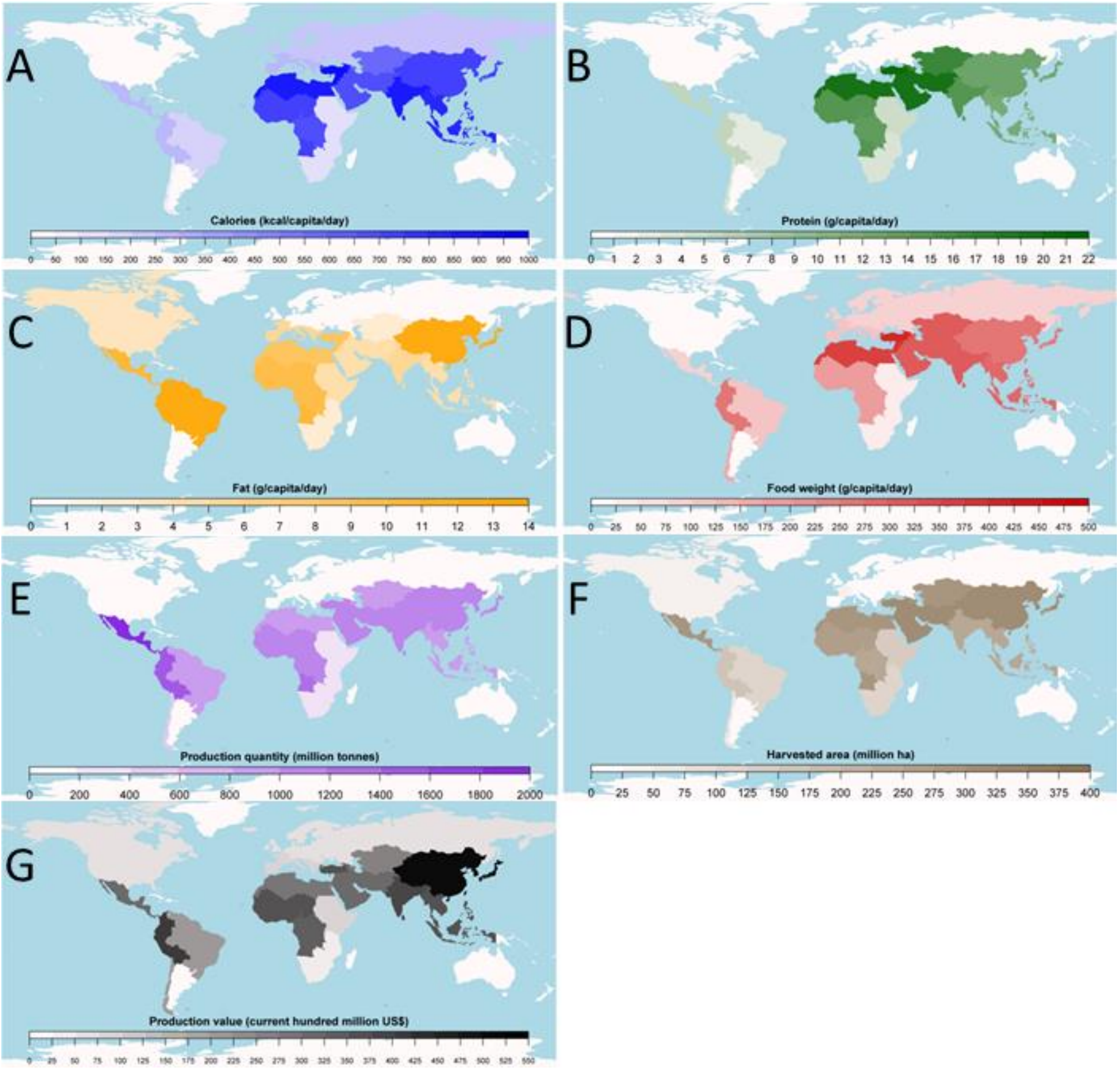

Figure S4. Importance of primary regions of diversity of crops in contribution to global aggregate food supplies [(A) calories, (B) protein, (C) fat, and (D) food weight] and total global agricultural production [(E) production quantity, (F) harvested area, and (G) production value], averaged over 2009-2011.

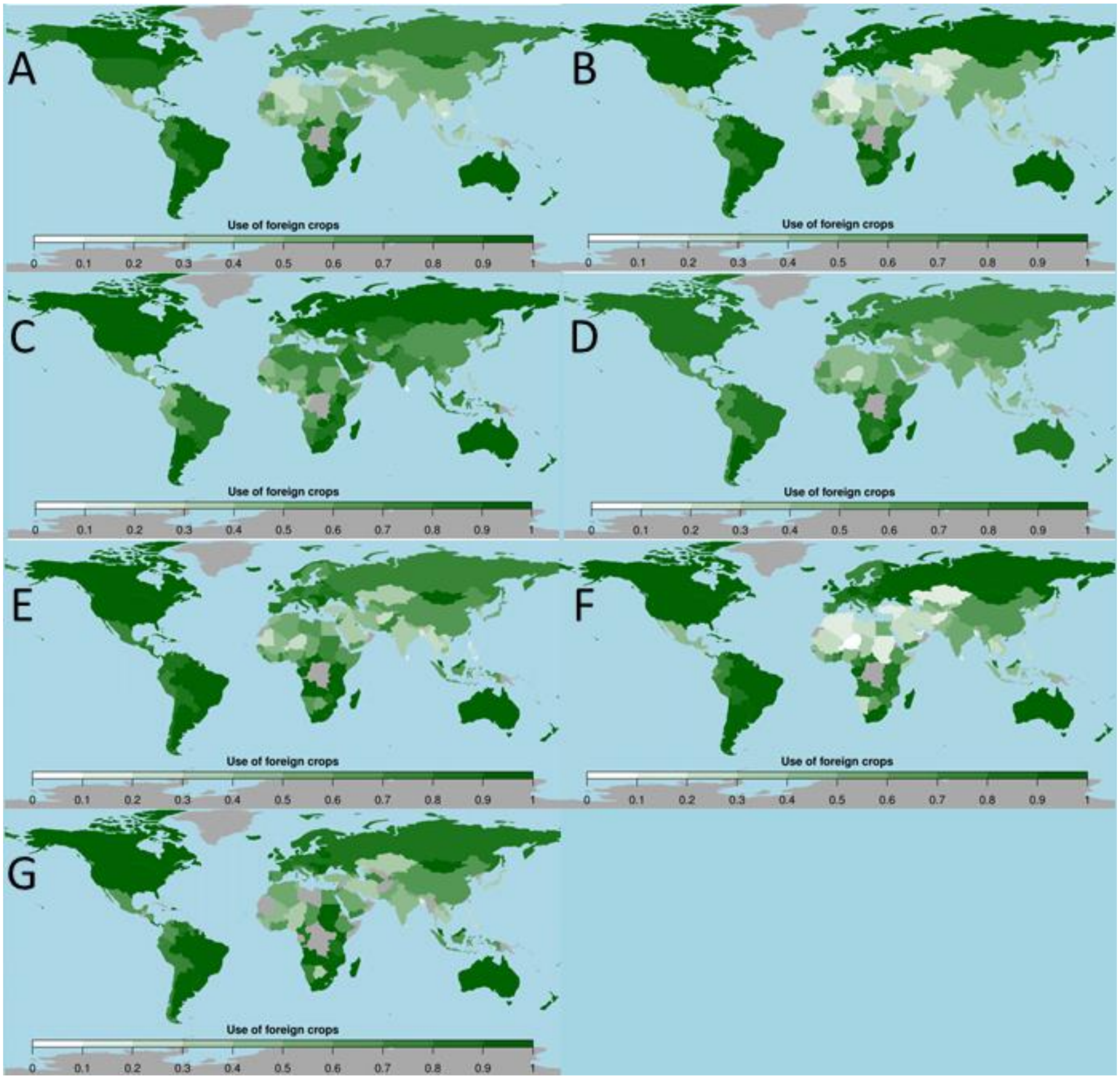

Figure S5. Degree of use of foreign crops per country in national food supplies [(A) calories, (B) protein, (C) fat, and (D) food weight] and national agricultural production systems [(E) production quantity, (F) harvested area, and (G) production value] as a modeled mean between minimum and maximum foreign crop use per country, 2009-2011. Scale is degree of use of foreign crops (1 = 100% use of foreign crops).

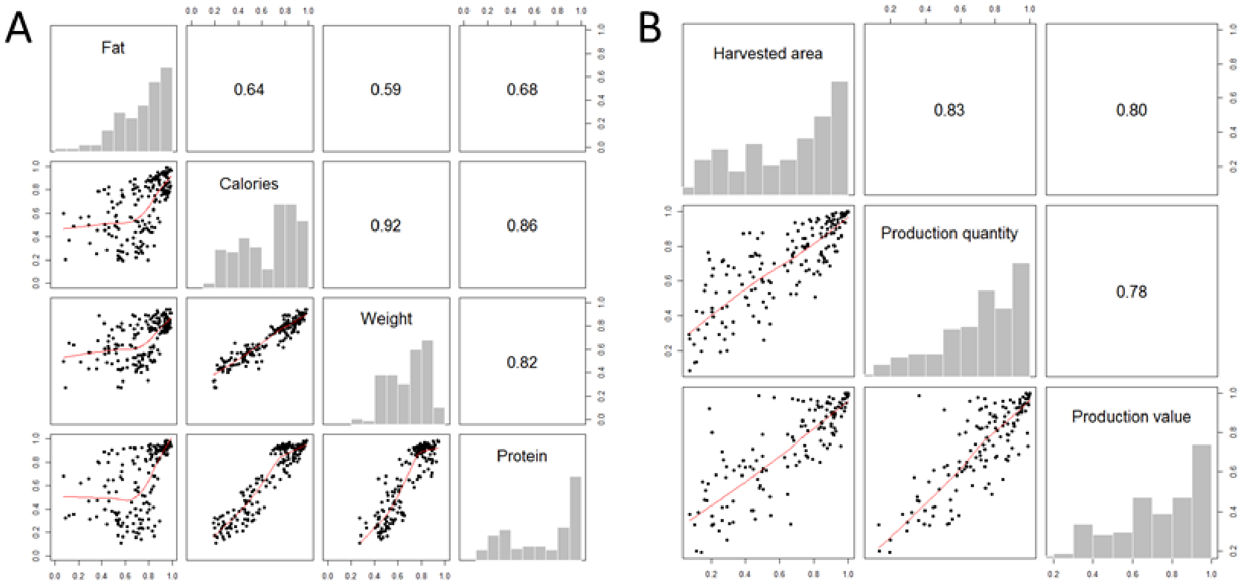

Figure S6. Correlation within countries in regard to use of foreign crops in (A) national food supplies, or (B) national agricultural production systems variables as a modeled mean between minimum and maximum use of foreign crops per country, 2009-2011. Scale is degree of use (1 = 100% use of foreign crops).

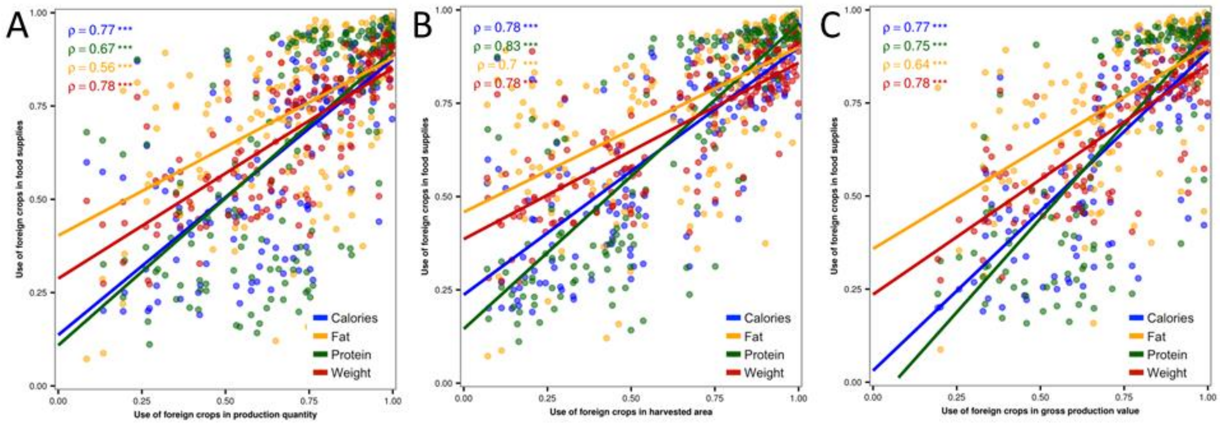

Figure S7. Correlation within countries in regard to use of foreign crops between national food supplies and national agricultural production [(A) production quantity, (B) harvested area, and (C) gross production value] variables, as a modeled mean between minimum and maximum use of foreign crops per country, 2009-2011. Scale is degree of use (1 = 100% use of foreign crops).

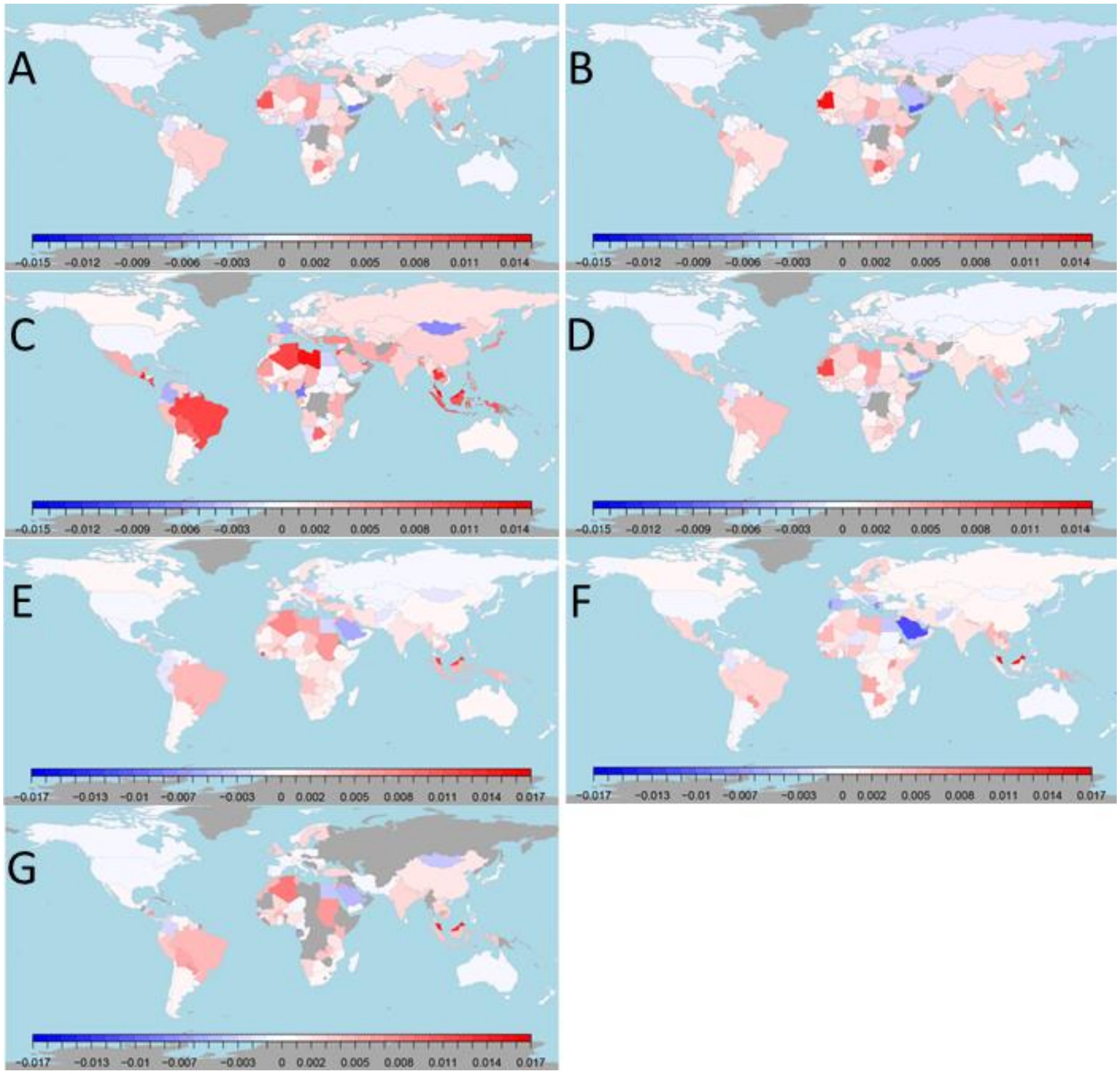

Figure S8. Slope of change in use of foreign crops from 1961 to 2009 in regard to national food supplies [(A) calories, (B) protein, (C) fat, and (D) food weight] and from 1961 to 2011 for national agricultural production systems [(E) production quantity, (F) harvested area, and (G) production value, measured as change in the modeled mean value between minimum and maximum use of foreign crops for each country in each year for each variable.

SI 2. Supplementary Tables

All supplementary tables are available on Dryad at doi:10.5061/dryad.s08t2

Table S1. Crop commodities assessed in food supplies and agricultural production systems analyses and their primary regions of diversity. Taxonomy follows GRIN (2015) [25].

Table S2. Countries assessed in food supplies and agricultural production systems analyses and their associated regions.

Table S3. Importance of primary regions of diversity of agricultural crops in contribution to national food supplies [as measured in contribution of crops to calories (kcal/capita/day), protein (g/capita/day), fat (g/capita/day), and food weight (g/capita/day)] and national agricultural production [production quantity (tonnes), harvested area (ha), and production value (million US$)], averaged over years 2009-2011. Importance was estimated by grouping the contribution of consumed/produced crops by their primary regions of diversity. As some crops pertain to more than one primary region of diversity, total values across all primary regions per country is not equivalent to total per capita food supply/ total agricultural production values per country. Percentages provide a comparison of the relative importance of primary regions in contribution to the food supply/national production of each country.

Table S4. Importance of primary regions of diversity of agricultural crops in contribution to regional food supplies [as measured in contribution of crops to calories (kcal/capita/day), protein (g/capita/day), fat (g/capita/day), and food weight (g/capita/day),] and total regional agricultural production [production quantity (tonnes), harvested area (ha), and production value (million US$)], averaged over years 2009-2011. Regional food supplies values (kcal or g, /capita/day) were formed by deriving a population-weighted average of national food supplies values across countries comprising each region. Regional production values were formed by summing national production values across countries comprising each region. Importance was estimated by grouping the contribution of consumed/produced crops by their primary regions of diversity. As some crops pertain to more than one primary region of diversity, total values across all primary regions per consuming/producing region is not equivalent to total per capita food supply/ total agricultural production values per consuming/producing region. Percentages provide a comparison of the relative importance of primary regions in contribution to the food supply/total production of each region.

Table S5. Crop commodity composition of regional food supplies [as measured in contribution of crops to calories (kcal/capita/day), protein (g/capita/day), fat (g/capita/day), and food weight (g/capita/day),] and total regional agricultural production [production quantity (tonnes), harvested area (ha), and production value (million US$)], averaged over years 2009-2011. Regional food supplies values (kcal or g, /capita/day) were formed by deriving a population-weighted average of national food supplies values across countries comprising each region. Regional production values were formed by summing national production values across countries comprising each region.

Table S6. Estimated percent use of foreign crops in current national food supplies and agricultural production systems. Data includes the raw mean minimum and maximum use values across years 2009-2011 per country, and the mean value between minimum and maximum per

country across these years, as well as modeled mean values and variation metrics as estimated in a Bayesian framework using an interval-censored response variable bounded between minimum and maximum use estimates.

Table S7. Change in use of foreign crops in national food supplies, from years 1961-2009. Data includes minimum and maximum use values for each variable for each country in each year, as well as slopes of change and variation metrics over the time period as estimated in a Bayesian framework using an interval-censored response variable bounded between minimum and maximum use estimates. Year was centered at 1985 for modeling purposes, thus model intercepts represent mean use in this year.

Table S8. Change in use of foreign crops in national agricultural production systems, from years 1961-2011. Data includes minimum and maximum use values for each variable for each country in each year, as well as slopes of change and variation metrics over the time period as estimated in a Bayesian framework using an interval-censored response variable bounded between minimum and maximum use estimates. Year was centered at 1985 for modeling purposes, thus model intercepts represent mean use in this year.
